# Association between Hyperuricemia and Hearing Impairment: Results from the Korean National Health and Nutrition Examination Survey

**DOI:** 10.3390/medicina59071273

**Published:** 2023-07-09

**Authors:** Hyemin Jeong, Young-Soo Chang, Chan-Hong Jeon

**Affiliations:** 1 Department of Internal Medicine, Division of Rheumatology, Soonchunhyang University Bucheon Hospital, Bucheon 14854, Republic of Korea; fly707@gmail.com; 2 Department of Otorhinolaryngology-Head and Neck Surgery, Sanggye Paik Hospital, College of Medicine, Inje University, Seoul 01757, Republic of Korea; yschang83@gmail.com

**Keywords:** uric acid, gout, hearing impairment

## Abstract

*Background and Objectives:* Hyperuricemia is associated with a variety of comorbidities. The objective of this study was to investigate the association between hyperuricemia and hearing impairment in Korean adults. *Materials and Methods:* Audiometric and laboratory test data from the 2019 to 2020 Korean National Health and Nutrition Examination Survey (KNHANES) were used for analysis. Hearing impairment was defined as a pure-tone average (0.5, 1, 2, 4 kHz) threshold level ≥ 41 decibels. The definition of hyperuricemia was different for males and females: >7 mg/dL for males vs. >6 mg/dL for females. *Results:* A total of 4857 (weight *n* = 17,990,725) subjects were analyzed. The mean age was 56.8 years old. The weighted prevalence was 12.1% for hyperuricemia and 2.5% for gout. The prevalence of hearing impairment was 13.4%. In the univariable analysis, hyperuricemia was significantly associated with hearing impairment. However, the diagnosis of gout was not associated with hearing impairment. In the multivariable analysis, hyperuricemia (odds ratios (OR): 1.41, 95% confidence interval [CI]: 1.03–1.92, *p* = 0.030) was associated with hearing impairment along with age (OR: 1.12, 95% CI: 1.10–1.14, *p* < 0.001), female sex (OR: 0.43, 95% CI: 0.34–0.64, *p* < 0.001), education (OR: 0.43, 95% CI: 0.30–0.63, *p* = 0.001), and occupational noise exposure (OR: 1.67, 95% CI: 1.25–2.22, *p* = 0.001). In the subgroup analysis, hyperuricemia was associated with hearing impairment in females (OR: 1.59, 95% CI: 1.02–2.48, *p* = 0.041) and the elderly aged 60 years or more (OR: 1.45, 95% CI: 1.05–1.99, *p* = 0.023). *Conclusions*: Hyperuricemia was independently associated with hearing impairment, especially in females and the elderly aged 60 years or more.

## 1. Introduction

Hearing loss is one of the most prevalent health conditions that people experience today. According to the World Health Organization (WHO), as of 2021, 430 million people had some degree of impaired hearing requiring hearing rehabilitation. It has been estimated that over 700 million people will have disabling hearing loss by 2050. Hearing impairment can have a significant impact on social interaction and the overall quality of life. Age-related hearing loss is associated with cognitive decline, cognitive impairment, and dementia [1]. Several factors are associated with the development of hearing impairment, including old age, male sex, smoking, noise exposure, and cardiovascular risks [2].

Uric acid is a metabolic product of purine nucleotides. Several components can affect serum uric acid levels, including dietary or lifestyle factors, drugs, and kidney function.

Gout is regarded as an arthritic condition resulting from the deposition of monosodium urate crystals in joints following chronic elevation of the uric acid level. Hyperuricemia and gout are associated with a variety of comorbidities, including chronic kidney disease, hypertension, obesity, heart failure, diabetes, myocardial infarction, and stroke [3]. Moon et al. have reported that a high serum uric acid level is one of the risk factors for age-related hearing impairment in adults aged 40 years or older who have undergone a comprehensive regular health checkup [4]. Recently, a large cohort study reported that gout is significantly associated with a 44% higher risk of new hearing loss in adults aged ≥65 years [5]. However, US National Health and Nutrition Examination Survey data have shown that higher uric acid levels are associated with better hearing sensitivity [6]. Thus, results about the association between uric acid and hearing impairment are inadequate and controversial. In addition, not all hyperuricemia presents as gout. If gout is diagnosed, medication to control uric acid might be taken. It is currently unclear whether hyperuricemia and gout are associated with hearing impairment.

The Korea National Health and Nutrition Examination Survey (KNHANES) is a nationwide survey assessing the general health and nutrition status of the South Korean population through interviews about health and nutrition and basic health assessments. In 2019–2020, the survey was conducted with a basic assessment of gout and serum uric acid levels. The aim of the present study was to evaluate the association between hyperuricemia/gout and hearing impairment by adjusting for various possible factors associated with hearing impairment in the Korean adult population using data from the 2019–2020 KNHANES. 

## 2. Materials and Methods

### 2.1. Study Population

Audiometric and laboratory test data from the 2019 to 2020 Korean National Health and Nutrition Examination Survey (KNHANES) were used for analysis. The methodology of KNHANES has been described in a previous study [7]. In brief, the KNHANES is a nationwide survey conducted periodically by the Korea Centers for Disease Control and Prevention to investigate the health and nutritional status of the Korean population. Using a proportional-allocation systematic sampling method with multistage stratification, the survey provides the power of sample weight to estimate characteristics of the entire population through a representative study group. Among the 15,469 participants in the 2019–2020 KNHANES, 6173 participants younger than 40 years were excluded because audiometric measurement was performed for participants aged 40 years or older in this survey. Among the remaining 9246 participants, 4439 had missing values for independent variables or outcome variables and were excluded. Finally, a total of 4857 (weighted *n* = 17,990,725) participants were selected for analysis.

### 2.2. Data Collection

To collect information on demographic variables, a standardized interview was performed individually by a professional investigator using an established questionnaire that consisted of demographic and socioeconomic characteristics. In detail, the questionnaire included data on age, sex, smoking status, alcohol consumption, body mass index (BMI), educational level, and occupational noise exposure. *Heavy alcohol use* was defined as the consumption of alcohol more than two or three times per week in the year prior to the interview. *Occupational noise exposure* was defined as a history of more than three years of exposure to loud noises such as machines or generators in the workplace. Loud noise means that the noise in the workplace is so loud that conversation is impossible. Gout was defined in the questionnaire as “Gout diagnosed by a physician (yes/no) through a standardized interview”. The question was, “Was your gout diagnosed by a physician?” Hypertension was defined as a systolic blood pressure of 140 mmHg or higher or a diastolic blood pressure of 90 mmHg or higher. Participants taking antihypertensive medication were also included in this group. Pre-hypertension was defined as a systolic blood pressure of 120–139 mmHg and a diastolic blood pressure of 80–89 mmHg. Diabetes was defined as a fasting blood glucose level of 126 mg/dL or higher, a diagnosis of diabetes by a physician, the use of hypoglycemic drugs or insulin injections, or a HbA1c level of 6.5% or higher. Pre-diabetes was defined as a fasting blood glucose level of 100–125 mg/dL or an HbA1c of 5.7–6.4%. Dyslipidemia was defined as a total cholesterol level greater than 240 mg/dL or the use of cholesterol-lowering drugs. The definition of hyperuricemia was different for males and females: >7 mg/dL for males and >6 mg/dL for females. Obesity was defined as a BMI of 25 or higher. Blood samples were collected in the morning after fasting for at least 8 h. Blood samples were immediately refrigerated and transported in cold storage to the central testing facility. Estimated glomerular filtration rate (eGFR) was calculated using the Modification of Diet in Renal Disease (MDRD) study equation: eGFR (mL/min/1.73 m^2^) = 175 × (serum creatinine) − 1.154 × (age) − 0.203 × (0.742 if female) [8]. This study was approved by the Institutional Review Board of Soonchunhyang University Hospital (IRB No. 2023-04-002).

### 2.3. Audiometric Measurement

A pure-tone air-conduction threshold was obtained in a soundproof booth using an automatic audiometer (AD629; Interacoustics, Denmark). The soundproof booth and audiometer were installed in the mobile vehicles, and the maximum permissible noise of the soundproof booth was strictly controlled under the regulation of ANSI-ASA S3.1. Trained professional investigators collected data independently for each ear at the following five frequencies: 0.5, 1.0, 2.0, 4.0, and 8.0 kHz. Hearing impairment was determined according to three categories of frequency (average, low/mid, high). Average-frequency pure-tone threshold was defined as the average of 0.5, 1.0, 2.0, and 4.0 kHz. Low/mid-frequency pure-tone threshold was defined as the average of air-conduction hearing thresholds measured at 0.5, 1.0, and 2.0 kHz. High-frequency pure-tone threshold was defined as the average of air-conduction hearing thresholds measured at 4.0 and 8.0 kHz. Hearing impairment was defined as a pure-tone average (0.5, 1, 2, 4 kHz) threshold level ≥41 decibels for the superior ear.

### 2.4. Statistical Analyses

Owing to sex differences in serum uric acid levels, analyses were stratified by sex. Potential factors associated with hyperuricemia, including age, sex, education level, current smoking, heavy alcohol use, occupational noise exposure, BMI, hypertension, diabetes, dyslipidemia, and eGFR *<* 60 mL/min/1.73 m^2^, were evaluated using a univariable analysis. Only variables with a *p*-value ≤ 0.2 were selected for the multivariable analysis with the logistic regression model. In the logistic regression analysis, we calculated adjusted odds ratios (ORs) with 95% confidence intervals (CIs) for hearing impairment. A subgroup analysis with stratification according to sex and age was also performed. Using the Bonferroni method, *p*-values were corrected. Statistical analyses were performed using SAS version 9.4 (SAS Institute, Cary, NC, USA). All *p*-values were two-sided, and *p*-values *<* 0.05 were considered statistically significant.

## 3. Results

### 3.1. Demographic and Clinical Characteristics of the Study Population

A total of 4857 adults aged ≥ 40 years (weighted *n* = 17,990,725) were analyzed for this study. The weighted demographic and clinical characteristics of the study population are presented in Table 1. The mean age of the participants was 56.8 years. Males accounted for 48.3%. The prevalence rates of current smoking and occupational noise exposure were 17.1% and 16.4%, respectively. The prevalence rates of diabetes, dyslipidemia, and hypertension were 18.5%, 31.4%, and 37.7%, respectively. Of all participants, 2.2% were diagnosed with gout by physicians. The prevalence of gout was 2.5%, and the prevalence of hyperuricemia (>7 mg/dL in males and >6 mg/dL in females) was 12.1%.

### 3.2. Association between Hyperuricemia/Gout and Hearing Impairment

To evaluate whether hyperuricemia and gout were associated with hearing impairment, a univariable logistic analysis was performed (Table 2). Gout did not show any significant association with hearing impairment (OR: 0.83, *p* = 0.554), whereas hyperuricemia did (OR: 1.55, *p* = 0.002). When subjects were divided into subgroups by age and sex, gout did not show any significant association in any subgroup analysis. Hyperuricemia showed a significant association with hearing impairment in females (OR: 2.71, *p* < 0.001) and the elderly aged ≥ 60 years (OR: 1.7, *p* = 0.002). Based on these results, we performed a multivariate analysis by selecting hyperuricemia as an independent variable associated with hearing impairment. 

### 3.3. Clinical Characteristics According to Presence of Hyperuricemia

Clinical characteristics were analyzed according to the presence of hyperuricemia (Table 3). Hyperuricemia was associated with a high BMI and decreased eGFR in both males and females.

Female subjects with hyperuricemia were older than those with normal uric acid levels. The ratio of education level according to the presence of hyperuricemia was significantly different among female subjects but not among male subjects. In addition, the weighted prevalence rates of diabetes and hypertension were higher in female subjects with hyperuricemia than in subjects with normal uric acid levels.

### 3.4. Factors Associated with Hearing Impairment

In the univariable logistic regression analysis for hearing impairment, age, sex, education level, occupational noise exposure, diabetes, dyslipidemia, hypertension, eGFR < 60 mL/min/1.73 m^2^, and hyperuricemia were associated with hearing impairment (Table 4). These potential associated factors and demographic variables, as well as smoking, alcohol consumption, and BMI, were included in the multivariable analysis with the logistic regression model.

In the multivariable-adjusted analysis, age (OR: 1.12, 95% CI: 1.1 to 1.14, *p* < 0.001), female sex (OR: 0.46, 95% CI: 0.34 to 0.64, *p* < 0.001), university-level education (OR: 0.43, 95% CI: 0.30 to 0.63, *p* < 0.001), occupational noise exposure (OR: 1.65, 95% CI: 1.25 to 2.22, *p* < 0.001), and hyperuricemia (OR: 1.41, 95% CI: 1.03 to 1.92, *p* = 0.031) were significantly associated with hearing impairment (Table 5). In the subgroup analysis according to sex, age (OR: 1.12, 95% CI: 1.12 to 1.14, *p* < 0.001), university-level education (OR: 0.61, 95% CI: 0.38 to 0.98, *p* < 0.040), and occupational noise exposure (OR: 1.91, 95% CI: 1.37 to 2.69, *p* < 0.001) were significantly associated with hearing impairment in male participants. Hyperuricemia was not significantly associated with hearing impairment in male participants. In the female subgroup analysis, hyperuricemia remained a significant factor associated with hearing impairment (OR: 1.59, 95% CI: 1.02 to 2.48, *p* = 0.041), age (OR: 1.11, 95% CI: 1.09 to 1.14, *p* < 0.001), and university-level education (OR: 0.33, 95% CI: 0.16 to 0.68, *p* = 0.003). Hyperuricemia was also significantly associated with hearing impairment in participants aged ≥ 60 years (OR: 1.45, 95% CI: 1.05 to 1.99, *p* = 0.023).

### 3.5. Factors Associated with Hearing Impairment According to Frequency

In the multivariable logistic regression analysis for high-frequency hearing impairment, age (OR: 1.16, 95% CI: 1.14 to 1.17, *p* < 0.001), female sex (OR: 0.25, 95% CI: 0.18 to 0.33, *p* < 0.001), university-level education (OR: 0.39, 95% CI: 0.28 to 0.54, *p* < 0.001), occupational noise exposure (OR: 1.65, 95% CI: 1.28 to 2.14, *p* < 0.001), and diabetes (OR: 1.31, 95% CI: 1.03 to 1.67, *p* = 0.032) were associated with high-frequency hearing impairment (Supplementary Table S1). Hyperuricemia was associated with high-frequency hearing impairment in participants aged ≥ 60 years in the subgroup analyses (OR: 1.85, 95% CI: 1.12 to 3.09, *p* = 0.018).

In the multivariable logistic regression analysis for low/mid-frequency hearing impairment, age (OR: 1.14, 95% CI: 1.12 to 1.16, *p* < 0.001), female sex (OR: 0.70, 95% CI: 0.49 to 0.98, *p* = 0.040), university-level education (OR: 0.42, 95% CI: 0.28 to 0.62, *p* < 0.001), and occupational noise exposure (OR: 1.93, 95% CI: 1.49 to 2.50, *p* < 0.001) were associated with low/mid-frequency hearing impairment (Supplementary Table S2). Hyperuricemia was not associated with low/mid-frequency hearing impairment in any subgroup analysis (male, female, age < 60 years, or age ≥ 60 years).

## 4. Discussion

This study has demonstrated that hyperuricemia is associated with hearing impairment using a national representative survey of the Korean adult population aged 40 years or older. Previous studies have reported that both hyperuricemia and gout can be associated with the development of hearing loss [5,6]. Herein, we have analyzed which of these two factors is significantly associated with hearing loss. Not all hyperuricemia cases present as gout, and not all gout patients present hyperuricemia due to uric acid-lowering therapy. We found an independent association between hyperuricemia and hearing impairment. However, the diagnosis of gout did not show an association with hearing impairment. These results indicate that hyperuricemia can increase the risk of hearing loss, regardless of the presence of gout. The results of this study suggest that controlling serum uric acid levels is more important than the presence or absence of a gout diagnosis in patients with hearing impairment.

It is still not well elucidated how increased uric acid levels can result in hearing impairment. One possible mechanism by which hyperuricemia may contribute to hearing impairment is through the accumulation of uric acid, which can cause vascular compromise, inflammation, and oxidative stress to the cochlea [9,10,11].

Uric acid can cause endothelial dysfunction by inhibiting proliferative effects on the inner lining of blood vessels and reducing the production of nitric oxide [11]. Uric acid is linked to the lysosomal membrane via a hydrogen bond, causing membrane lysis [12]. Hyperuricemia is also associated with the activation of circulating platelets, which can also result in endothelial dysfunction [13]. Considering that the cochlea has a single arterial supply and is highly sensitive to changes in blood flow [14], degenerative vascular diseases might cause subclinical damage to the cochlea with blood flow reduction or fluctuation in the cochlea.

Another possible mechanism is uric acid-induced systemic inflammation. An Italian cohort study of people aged 65 to 95 years revealed that uric acid is significantly associated with increased neutrophil count, C-reactive protein, IL-6, IL-18, and TNFα [15]. Another population-based study performed in Switzerland in people aged 35 to 75 years reported that uric acid is positively associated with CRP, TNFα, and IL-6 but negatively associated with IL-1β, particularly in females [16]. Hyperuricemia can induce monosodium urate (MSU) crystal formation. It is well established that MSU crystals can induce the inflammatory process through Toll-like receptors, which can result in the activation of the NALP3 inflammasome and proinflammatory cytokines such as interleukin (IL)-1β [17]. Uric acid can stimulate the production of IL-1β, IL-6, and the tumor necrosis factor (TNF)-α in human mononuclear cells [18]. The in vivo production of TNF-α, IL-1β, and IL-6 in the cochlea along with synergic leukocyte infiltration has been well described in experimental studies on inner ear inflammation [19,20]. The immune responses occurring in the cochlea, including the recruitment of many immune cells, can induce leukocyte infiltration, scar formation, and gliosis. These changes are linked to immune-mediated hearing loss. Therefore, increased serum uric acid levels might play a role in the systemic inflammatory process, including in the cochlea.

Uric acid can mediate the generation of free radicals and serve as a pro-oxidant. Uric acid can induce oxidative stress in human vascular cells by inducing reactive oxygen species (ROS) production [21]. ROS are unstable molecular species that contain one or more unpaired electrons, which makes them highly reactive [22]. As a consequence of the imbalance between the production of free radicals and endogenous antioxidant systems, ROS concentrations might increase, leading to toxicity and causing oxidative stress-induced cell damage [23]. It is well established that an excess of free radicals in the cochlear sensory epithelium, the spiral ganglion neurons, and the cells of the stria vascularis is a key factor in the pathogenesis of cell damage in the cochlea, which can result in the development of sensorineural hearing loss [24,25]. However, uric acid can also serve as an antioxidant. Uric acid can scavenge free radicals in human serum [26]. Free-radical scavenging capacity is increased in healthy participants with systemic uric acid administration [27]. This dual function of pro-oxidant and antioxidant might be regulated by a molecular switch in different compartments of our body [28]. Considering that ROS overproduction is one of the most promising therapeutic strategic targets in otologic fields [29], further research studies should be performed to determine whether hyperuricemia might be a potentially useful therapeutic target for controlling ROS in the cochlea.

In this study, hypruricemia was associated with high-frequency hearing impairment in older adults. It corresponded well with a previous study reporting significant abnormalities in otoacoustic emissions at higher frequencies (4 and 5 kHz) in participants with hyperuricemia [30]. High-frequency hearing loss has been detected in patients with gout [31]. Considering that microvascular degenerative diseases with various etiologies can cause cochlear dysfunction, especially in high-frequency bands of the cochlea [32,33,34], our results might shed additional light on the association between hyperuricemia and high-frequency hearing loss.

In the subgroup analysis, hyperuricemia showed a significant association with the female gender. In females, serum uric acid levels are lower than in males. However, uric acid levels increase after menopause [35]. Estrogen can affect uric acid metabolism and renal clearance [36]. Hyperuricemia is significantly associated with decreased lung function in females but not in males [7]. Serum uric acid is more closely associated with metabolic syndrome in females than in males [37]. Hyperuricemia can increase the risk of cardiovascular disease mortality by 67% in women but not in men [38]. Taken together, the relative physiologic impact of hyperuricemia seems to be stronger in females than in males.

A number of studies have demonstrated that hyperuricemia is linked to cardiovascular and neurologic diseases, including hypertension, coronary heart disease, metabolic syndrome, stroke, and neurodegenerative diseases [39]. It has been reported that hyperuricemia is associated with higher reported risks of all-cause and cardiovascular disease mortality in the Taiwanese general population [40]. In a case-matched cohort study, hyperuricemia patients without gout who received uric acid-lowering therapy had potentially better survival than patients who did not receive uric acid-lowering therapy [41]. The current guidelines do not recommend initiating uric acid-lowering therapy in patients with asymptomatic hyperuricemia because the benefits of uric acid-lowering therapy would not outweigh the potential treatment costs or risks for a large number of patients [42,43]. It is deemed that regulating uric acid levels in individuals who are indicated for uric acid-lowering therapy might be beneficial for reducing the risk of comorbidities and maintaining auditory function. Further studies are needed to determine the possible role of uric acid-lowering therapy in protecting hearing loss in patients with hyperuricemia.

This study has several limitations. First, this study was unable to obtain information on uric acid-lowering agents or diuretics that might have an impact on serum uric acid levels. Second, the diagnosis of gout was dependent on the information provided by the participants in the interview. In this study, specific criteria were not applied for the definition of gout. Third, audiometric measurements were only performed for participants aged 40 years or older in this survey. Considering age is an important factor associated with hearing impairment, the results of the study should be interpreted only for individuals aged 40 years or older. Finally, it was not possible to establish causality due to the cross-sectional design of this study. Despite all these limitations, the strength of this study lies in the fact that we analyzed the relationship between uric acid and hearing impairment in a nationwide representative sample of the general adult population. An audiometric test was conducted, and analyses were performed to determine whether gout or uric acid levels were associated with hearing.

## 5. Conclusions

In conclusion, the current study highlights that hyperuricemia is independently associated with hearing impairment, especially in females and older adults (≥60 years). Further investigations are required to clarify the relationship between serum uric acid levels and hearing impairment.

## Figures and Tables

**Table 1:** Demographic and clinical characteristics of the study population (unweighted, *n* = 4857; weighted, *n* = 17,990,725).

Variable	Category	N (Weighted %)
Age, years		56.8 ± 0.3
Sex	Male	8,691,336 (48.3)
	Female	9,299,389 (51.7)
Education level	Elementary school	2,995,942 (16.7)
	Middle school	1,981,792 (11.0)
	High school	6,338,706 (35.2)
	University	6,674,285 (37.1)
Marital status	Married	17,155,817 (95.4)
	Unmarried	834,908 (4.6)
Current smoking	Never smoking	10,145,665 (56.4)
	Ex-smoker	4,761,340 (26.5)
	Current smoker	3,083,720 (17.1)
Heavy alcohol use *	No	13,951,063 (77.6)
	Yes	4,039,662 (22.5)
Occupational noise exposure ^†^	No	15,044,870 (83.6)
	Yes	2,945,855 (16.4)
Body mass index (kg/m^2^)	BMI < 25	10,899,796 (60.6)
	BMI ≥ 25	7,090,929 (39.4)
Diabetes	Normal	6,240,013 (34.7)
	Pre-diabetes	8,421,703 (46.8)
	Diabetes	3,329,009 (18.5)
Dyslipidemia	No	12,349,295 (68.6)
	Yes	5,641,430 (31.4)
Hypertension	Normal	6,293,940 (35.0)
	Pre-hypertension	4,918,288 (27.3)
	Hypertension	6,778,497 (37.7)
Gout	No	17,534,556 (97.5)
	Yes	456,169 (2.5)
Systolic blood pressure, mmHg		121.2 ± 0.4
Diastolic blood pressure, mmHg		77.2 ± 0.2
Total cholesterol, mg/dL		193.5 ± 0.8
High-density cholesterol, mg/dL		50.9 ± 0.3
Triglyceride, mg/dL		141.9 ± 2.5
Low-density cholesterol, mg/dL		114.3 ± 0.8
Aspartate transaminase, IU/L		25.2 ± 0.2
Alanine transaminase, IU/L		23.9 ± 0.3
Uric acid, mg/dL		5.09 ± 0.02
Hyperuricemia ^‡^	No	15,813,858 (87.9)
	Yes	2,176,886 (12.1)
BUN, mg/dL		15.6 ± 0.1
Serum creatinine, mg/dL		0.82 ± 0.00
eGFR, mL/min/1.73 m^2^	eGFR < 60	722,575 (4.0)
	eGFR ≥ 60	17,268,150 (96.0)

Continuous variables are expressed as mean ± standard error of the mean. eGFR, estimated glomerular filtration rate; BUN, blood urea nitrogen. * Defined as the consumption of alcohol more than two or three times per week in the year prior to the interview. ^†^ Defined as a history of more than 3 years of exposure to loud noise at work. Loud noise means that the noise in the workplace is so loud that conversation is impossible. ^‡^ Defined as >7 mg/ dL in males and >6 mg/dL in females.

**Table 2:** Association analysis between hearing impairment * and hyperuricemia/gout in Korean adult population according to age and sex.

	Total		Sex				Age			
Variable			Male		Female		Age < 60 Years		Age ≥ 60 Years	
	Weighted OR (95% CI)	*p*-Value	Weighted OR (95% CI)	*p*-Value	Weighted OR (95% CI)	*p*-Value	Weighted OR (95% CI)	*p*-Value	Weighted OR (95% CI)	*p*-Value
Gout	0.83 (0.44, 1.55)	0.554	0.69 (0.35, 1.35)	0.282	0.94 (0.12, 7.43)	0.952	0.68 (0.16, 2.97)	0.611	0.83 (0.4, 1.73)	0.618
Hyperuricemia ^†^	1.55 (1.18, 2.03)	0.002	1.03 (0.72, 1.49)	0.856	2.71 (1.8, 4.09)	<0.0001	1.34 (0.68, 2.64)	0.405	1.7 (1.22, 2.38)	0.002

* Defined as a pure-tone average (0.5, 1, 2, 4 kHz) threshold level of ≥41 decibels for the superior ear; ^†^ defined as >7 mg/dL in males and >6 mg/dL in females; OR, odds ratio.

**Table 3:** Demographic and clinical characteristics according to the presence or absence of hyperuricemia in the Korean adult population.

		Male (*n* = 2118, Weighted *n* = 8,691,336)			Female (*n* = 2739, Weighted *n* = 9,229,389)		
Variable	Category	Normal UA UA < 7 mg/dL *n* = 1759 Weighted *n* = 7,187,399	Hyperuricemia UA ≥ 7 mg/dL *n* = 359 Weighted *n* = 1,503,936	*p*-Value	Normal UA UA < 6 mg/dL (*n* = 2524, Weighted *n* = 8,626,459)	Hyperuricemia UA ≥ 6 mg/dL (*n* = 215, Weighted *n* = 672,930)	*p*-Value
Age, years		56.6 ± 0.4	54.2 ± 0.7	<0.001	56.93 ± 0.37	62.92 ± 0.97	<0.001
Education level	Elementary school	800,625 (11.1)	155,701 (10.4)	0.935	1,767,698 (20.5)	271,918 (40.4)	<0.001
	Middle school	757,357 (10.5)	144,503 (9.6)		991,327 (11.5)	88,606 (13.2)	
	High school	2,473,190 (34.4)	514,109 (34.2)		3,188,732 (37.0)	162,676 (24.2)	
	University	3,156,227 (43.9)	689,624 (45.9)		2,678,703 (31.1)	149,731 (22.3)	
Current smoking	Never smoking	1,370,952 (19.1)	257,091 (17.1)	0.454	7,933,117 (92.0)	584,506 (86.8)	0.093
	Ex-smoker	3,481,894 (48.4)	798,304 (53.1)		419,409 (4.8)	61,733 (9.2)	
	Current smoker	2,334,554 (32.5)	448,541 (29.8)		273,934 (3.2)	26,691 (4.0)	
Heavy alcohol use *	No	4,626,014 (64.4)	933,307 (62.1)	0.508	7,799,737 (90.4)	592,005 (88.0)	0.343
	Yes	2,561,386 (35.6)	570,629 (37.9)		826,722 (9.6)	80,925 (12.0)	
Occupational noise exposure ^†^	No	5,568,403 (77.5)	1,235,270 (82.1)	0.066	7,644,186 (88.6)	597,013 (88.7)	0.970
	Yes	1,618,997 (22.5)	268,667 (17.9)		982,274 (11.4)	75,917 (11.3)	
Body mass index (kg/m^2^)	BMI < 25	4,036,396 (56.2)	625,657 (41.6)	<0.001	5,943,042 (68.9)	294,702 (43.8)	<0.001
	BMI ≥ 25	3,151,004 (43.8)	878,279 (58.4)		2,683,418 (31.1)	378,228 (56.2)	
Diabetes	Normal	2,213,239 (30.8)	393,456 (26.1)	0.046	3,515,586 (40.7)	117,732 (17.5)	<0.001
	Pre-diabetes	3,344,054 (46.5)	838,892 (55.8)		3,860,898 (44.8)	377,859 (56.2)	
	Diabetes	1,630,107 (22.7)	271,589 (18.1)		1,249,975 (14.5)	177,339 (26.3)	
Dyslipidemia	No	5,120,649 (71.2)	1,006,763 (66.9)	0.219	5,801,461 (67.3)	420,422 (62.5)	0.162
	Yes	2,066,751 (28.8)	497,173 (33.1)		2,824,998 (32.7)	252,508 (37.5)	
Hypertension	Normal	2,046,641 (28.5)	359,640 (23.9)	0.176	3,727,327 (43.2)	160,332 (22.8)	<0.001
	Pre-hypertension	2,205,245 (30.7)	438,879 (29.2)		2,141,151 (24.8)	133,014 (19.8)	
	Hypertension	2,935,514 (40.8)	705,417 (46.9)		2,757,981 (32.0)	379,585 (56.4)	
Systolic blood pressure, mmHg		121.8 ± 0.5	123.6 ± 1.0	0.092	119.77 ± 0.53	126.88 ±1.25	<0.001
Diastolic blood pressure, mmHg		78.9 ± 0.3	80.9 ± 0.8	0.012	75.3 ± 0.24	74.99 ±0.77	0.694
Total cholesterol, mg/dL		188.02 ± 1.3	199.07 ± 2.84	0.001	197.1 ± 1.1	194.9 ± 3.1	0.553
HDL, mg/dL		46.9 ± 0.3	44.9 ± 0.6	0.005	55.39 ± 0.33	48.3 ± 0.8	<0.001
Triglyceride, mg/dL		159.7 ± 4.5	210.4 ± 10.7	<0.001	114.2 ± 2.0	153.0 ± 6.7	<0.001
LDL, mg/dL		109.1 ± 1.4	112.1 ± 3.1	0.412	118.8 ± 0.9	116.1 ± 3.2	0.422
Aspartate transaminase, IU/L		26.3 ± 0.4	30.9 ± 1.4	0.001	23.2 ± 0.2	25.97 ± 1	0.011
Alanine transaminase, IU/L		27.3 ± 0.5	33.5 ± 1.4	<0.001	19.5 ± 0.3	23.39 ± 1.68	0.026
Uric acid, mg/dL		5.40 ± 0.03	7.77 ± 0.04	<0.001	4.24 ± 0.02	6.68 ± 0.05	<0.001
BUN, mg/dL		15.9 ± 0.1	16.6 ± 0.4	0.081	14.9 ± 0.1	18.6 ± 0.5	<0.001
Creatinine, mg/dL		0.93 ± 0	1.03 ± 0.02	<0.001	0.68 ± 0	0.89 ± 0.03	<0.001
eGFR, mL/min/1.73 m^2^	eGFR ≥60	6,957,257 (96.8)	1,341,368 (89.2)	<0.001	8,453,185 (98.0)	516,339 (76.7)	<0.001
	eGFR <60	230,142 (3.2)	162,568 (10.8)		173,274 (2.01)	156,591 (23.2)	

Continuous variables are expressed as mean ± standard error of the mean; * defined as the consumption of alcohol more than two or three times per week in the year prior to the interview; ^†^ defined as a history of more than 3 years of exposure to loud noise at work. Loud noise means that the noise in the workplace is so loud that conversation is impossible; UA, uric acid; BMI, body mass index; eGFR, estimated glomerular filtration rate; BUN, blood urea nitrogen; HDL, high-density lipoprotein; LDL, low-density lipoprotein.

**Table 4:** Univariable logistic regression analysis for predicting the risk of hearing impairment in the Korean adult population according to age and sex.

Variable	Category	Total		Male		Female		Age < 60 Years		Age ≥ 60 Years	
		Weighted OR (95% CI)	*p*-Value	Weighted OR (95% CI)	*p*-Value	Weighted OR (95% CI)	*p*-Value	Weighted OR (95% CI)	*p*-Value	Weighted OR (95% CI)	*p*-Value
Age, years		1.13 (1.11, 1.14)	<0.001	1.12 (1.1, 1.14)	<0.001	1.14 (1.12, 1.16)	<0.001	1.12 (1.08, 1.17)	<0.001	1.14 (1.12, 1.17)	<0.001
Sex (reference: male)	Female	0.70 (0.59, 0.83)	<0.001					0.44 (0.28, 0.71)	<0.001	0.67 (0.55, 0.82)	<0.001
Education level (reference: elementary)			<0.001		<0.001		<0.001		0.001		<0.001
	Middle school	0.44 (0.34, 0.57)	<0.001	0.72 (0.47, 1.10)	0.133	0.22 (0.15, 0.34)	<0.001	1.30 (0.51, 3.34)	0.587	0.53 (0.39, 0.7)	<0.001
	High school	0.25 (0.19, 0.33)	<0.001	0.38 (0.25, 0.56)	<0.001	0.14 (0.10, 0.19)	<0.001	0.91 (0.33, 2.48)	0.856	0.50 (0.38, 0.66)	<0.001
	University	0.09 (0.07, 0.13)	<0.001	0.12 (0.08, 0.18)	<0.001	0.05 (0.02, 0.09)	<0.001	0.25 (0.09, 0.67)	0.006	0.41 (0.31, 0.56)	<0.001
Current smoking (reference: never smoking)			0.059		0.203		0.001		0.004		0.235
	Ex-smoker	1.27 (1.04, 1.54)	0.020	1.08 (0.79, 1.47)	0.618	0.23 (0.09, 0.58)	0.002	2.07 (1.15, 3.74)	0.016	1.17 (0.92, 1.49)	0.198
	Current smoker	1.00 (0.76, 1.31)	0.976	0.81 (0.56, 1.18)	0.279	0.51 (0.23, 1.12)	0.097	1.93 (1.06, 3.51)	0.032	1.30 (0.89, 1.89)	0.179
Heavy alcohol use *	Yes	0.78 (0.60,1.00)	0.054	0.80 (0.60, 1.07)	0.134	0.19 (0.09, 0.42)	<0.001	1.27 (0.8, 2.03)	0.312	0.91 (0.68, 1.22)	0.536
Occupational noise exposure ^†^	Yes	1.69 (1.35, 2.17)	<0.001	1.85 (1.41, 2.50)	<0.001	1.20 (0.51, 1.82)	0.372	2.70 (1.69, 4.35)	<0.001	1.33 (1.02, 1.75)	0.039
Body mass index (kg/m^2^)	BMI ≥ 25	0.95 (0.78, 1.15)	0.589	0.87 (0.66, 1.14)	0.313	0.94 (0.72, 1.22)	0.652	1.25 (0.77, 2.02)	0.373	0.83 (0.65, 1.05)	0.115
Diabetes	Yes	2.38 (1.83, 3.09)	<0.001	1.70 (1.18, 2.46)	0.005	3.28 (2.26, 4.77)	<0.001	1.45 (0.74, 2.85)	0.285	1.17 (0.85, 1.62)	0.326
Dyslipidemia	Yes	1.42 (1.17, 1.72)	<0.001	1.29 (0.98, 1.70)	0.076	1.64 (1.26, 2.12)	<0.001	1.59 (1.03, 2.46)	0.039	0.87 (0.70, 1.08)	0.196
Hypertension	Yes	2.56 (2.00, 3.28)	<0.001	1.75 (1.29, 2.37)	<0.001	3.51 (2.40, 5.12)	<0.001	1.58 (0.91, 2.72)	0.103	1.29 (0.96, 1.72)	0.093
eGFR, mL/min/1.73 m^2^	eGFR < 60	4.00 (2.78, 5.88)	<0.001	3.45 (2.08, 5.88)	<0.001	4.76 (2.86, 8.33)	<0.001	3.13 (0.66, 14.29)	0.150	1.89 (1.25, 2.86)	0.003
Hyperuricemia ^‡^	Yes	1.55 (1.18, 2.03)	0.002	1.03 (0.72, 1.49)	0.856	2.71 (1.80, 4.09)	<0.001	1.34 (0.68, 2.64)	0.405	1.70 (1.22, 2.38)	0.002

* Defined as the consumption of alcohol more than two or three times per week in the year prior to the interview; ^†^ defined as a history of more than 3 years of exposure to loud noise at work. Loud noise means that the noise in the workplace is so loud that conversation is impossible; ^‡^ defined as >7 mg/dL in males and >6 mg/dL in females; OR, odds ratio; BMI, body mass index; eGFR, estimated glomerular filtration rate.

**Table 5:** Multivariable logistic regression analysis to predict risk of hearing impairment in the Korean adult population according to age and sex.

Variable	Category	Total		Male		Female		Age < 60 Years		Age ≥ 60 Years	
		Weighted OR (95% CI)	*p*-Value	Weighted OR (95% CI)	*p*-Value	Weighted OR (95% CI)	*p*-Value	Weighted OR (95% CI)	*p*-Value	Weighted OR (95% CI)	*p*-Value
Age, years		1.12 (1.1, 1.14)	<0.001	1.12 (1.1, 1.14)	<0.001	1.11 (1.09, 1.14)	<0.001				
Sex (reference: male)	Female	0.46 (0.34, 0.64)	<0.001					0.42 (0.26, 0.69)	<0.001	0.39 (0.29, 0.54)	<0.001
			<0.001		0.002		<0.001		<0.001		<0.001
Education level (reference: elementary)	Middle school	0.72 (0.54, 0.95)	0.020	1.28 (0.82, 2.01)	0.280	0.41 (0.26, 0.64)	<0.001	1.22 (0.46, 3.21)	0.690	0.50 (0.37, 0.67)	<0.001
	High school	0.85 (0.64, 1.15)	0.300	1.23 (0.79, 1.92)	0.353	0.62 (0.41, 0.94)	0.027	0.91 (0.32, 2.62)	0.862	0.44 (0.33, 0.59)	<0.001
	University	0.43 (0.3, 0.63)	<0.001	0.61 (0.38, 0.98)	0.040	0.33 (0.16, 0.68)	0.003	0.24 (0.08, 0.7)	0.010	0.33 (0.23, 0.47)	<0.001
			0.534		0.514		0.247		0.648		0.247
Current smoking (reference: never smoking)	Ex-smoker	0.9 (0.65, 1.23)	0.494	0.97 (0.67, 1.41)	0.879	0.42 (0.14, 1.2)	0.105	1.15 (0.64, 2.07)	0.625	0.76 (0.54, 1.07)	0.117
	Current smoker	1.11 (0.72, 1.7)	0.629	1.24 (0.76, 2.04)	0.384	0.8 (0.32, 2.02)	0.644	0.88 (0.46, 1.69)	0.706	0.75 (0.47, 1.2)	0.234
Heavy alcohol use *	Yes	0.91 (0.68, 1.21)	0.511	0.95 (0.69, 1.32)	0.773	0.52 (0.23, 1.2)	0.129	0.93 (0.59, 1.48)	0.772	0.72 (0.52, 1)	0.054
Occupational noise exposure ^†^	Yes	1.65 (1.25, 2.22)	<0.001	1.91 (1.37, 2.69)	<0.001	1.25 (0.79, 1.97)	0.344	1.89 (1.18, 3.02)	0.009	1.18 (0.88, 1.59)	0.265
Body mass index (kg/m^2^)	BMI ≥ 25	0.91 (0.72, 1.15)	0.429	1.07 (0.78, 1.47)	0.666	0.72 (0.51, 1.00)	0.054	1.00 (0.60, 1.66)	0.988	0.74 (0.58, 0.93)	0.012
Diabetes	Yes	0.96 (0.75, 1.21)	0.730	0.77 (0.56, 1.06)	0.116	1.13 (0.81, 1.57)	0.489	0.72 (0.38, 1.34)	0.300	1.13 (0.88, 1.45)	0.344
Dyslipidemia	Yes	1.04 (0.83, 1.3)	0.735	1.20 (0.86, 1.66)	0.288	0.9 (0.65, 1.23)	0.504	1.47 (0.94, 2.29)	0.090	0.89 (0.71, 1.12)	0.331
Hypertension	Yes	1.03 (0.8, 1.32)	0.836	1.09 (0.8, 1.47)	0.581	0.89 (0.62, 1.29)	0.554	1.19 (0.73, 1.94)	0.482	1.29 (0.99, 1.67)	0.056
eGFR, mL/min/1.73 m^2^	eGFR ≥ 60	1.06 (0.68, 1.67)	0.786	1.10 (0.60, 2.00)	0.768	1.00 (0.59, 1.69)	0.992	2.32 (0.56, 10.00)	0.253	1.37 (0.89, 2.08)	0.150
Hyperuricemia ^‡^	Yes	1.41 (1.03, 1.92)	0.031	1.34 (0.88, 2.06)	0.176	1.59 (1.02, 2.48)	0.041	0.99 (0.52, 1.89)	0.970	1.45 (1.05, 1.99)	0.023

* Defined as the consumption of alcohol more than two or three times per week in the year prior to the interview; ^†^ defined as a history of more than 3 years of exposure to loud noise at work. Loud noise means that the noise in the workplace is so loud that conversation is impossible; ^‡^ defined as >7 mg/dL in males and >6 mg/dL in females; OR, odds ratio; BMI, body mass index; eGFR, estimated glomerular filtration rate.

## Data Availability

All data and materials used and analyzed during the course of this study are available from the corresponding author upon reasonable request.

## Supplementary Materials

The following supporting information can be downloaded at https://www.mdpi.com/article/10.3390/medicina59071273/s1, Table S1: Multivariable logistic regression analysis to predict risk of high-frequency hearing impairment in the Korean adult population according to age and sex. Table S2: Multivariable logistic regression analysis to predict risk of low/mid-frequency hearing impairment in the Korean adult population according to age and sex.
