# Supplementary material for: Association between Hyperuricemia and Hearing Impairment: Results from the Korean National Health and Nutrition Examination Survey
Source: Medicina (Kaunas). 2023 Jul 9;59(7):1273. doi: 10.3390/medicina59071273 (PMC10385961; doi:10.3390/medicina59071273)
Supplement: Supplementary file 1 [file medicina-59-01273-s001.zip › medicina-2442429-supplementary.pdf]

Supplementary Table S1. Multivariable logistic regression analysis to predict risk of high frequency hearing impairment in the Korean adult population according to age and sex.

**Variable**	**category**	**Total**		**Male**		**Female**		**Age < 60 years**		**Age ≥ 60 years**	
		**Weighted OR (95% CI)**	**P-value**	**Weighted OR (95% CI)**	**P-value**	**Weighted OR (95% CI)**	**P-value**	**Weighted OR (95% CI)**	**P-value**	**Weighted OR (95% CI)**	**P-value**
Age, years		1.16 (1.14, 1.17)	< 0.001	1.16 (1.14, 1.17)	< 0.001	1.16 (1.14, 1.18)	< 0.001				
Sex (reference : male)	Female	0.25 (0.18, 0.33)	< 0.001					0.25 (0.18, 0.35)	< 0.001	0.17 (0.10, 0.29)	< 0.001
Education level (reference: Elementary)			< 0.001		0.006		< 0.001		< 0.001		< 0.001
	Middle school	0.76 (0.53, 1.08)	0.128	1.27 (0.64, 2.54)	0.493	0.63 (0.42, 0.94)	0.026	0.52 (0.24, 1.15)	0.107	0.55 (0.37, 0.83)	0.004
	High school	0.61 (0.45, 0.83)	0.002	1.09 (0.62, 1.89)	0.771	0.48 (0.33, 0.69)	< 0.001	0.28 (0.14, 0.56)	< 0.001	0.38 (0.27, 0.53)	< 0.001
	University	0.39 (0.28, 0.54)	< 0.001	0.68 (0.38, 1.21)	0.188	0.30 (0.20, 0.46)	< 0.001	0.15 (0.07, 0.29)	< 0.001	0.28 (0.19, 0.40)	< 0.001
Current s moking (reference: never smoking)			0.377		0.493		0.665		0.774		0.144
	Ex-smoker	0.98 (0.72, 1.31)	0.869	0.92 (0.65, 1.32)	0.663	0.99 (0.52, 1.89)	0.975	1.04 (0.74, 1.44)	0.837	0.62 (0.38, 1.02)	0.063
	Current smoker	1.2 (0.87, 1.65)	0.271	1.12 (0.77, 1.63)	0.561	1.37 (0.69, 2.69)	0.366	0.92 (0.64, 1.33)	0.655	0.87 (0.49, 1.55)	0.635
Heavy alcohol use[*]	Yes	1.11 (0.87, 1.42)	0.406	1.14 (0.86, 1.52)	0.352	1.00 (0.60, 1.66)	0.996	1.10 (0.86, 1.41)	0.435	0.68 (0.44, 1.04)	0.078
Occupational noise exposure[†]	Yes	1.65 (1.28, 2.14)	< 0.001	1.95 (1.34, 2.83)	0.001	1.23 (0.89, 1.71)	0.211	1.5 (1.07, 2.11)	0.019	1.57 (1.06, 2.33)	0.027
Body mass index (kg/m^2)	BMI≥25	1.00 (0.82, 1.21)	0.972	1.02 (0.78, 1.33)	0.885	0.93 (0.71, 1.22)	0.594	0.97 (0.77, 1.23)	0.824	0.78 (0.6, 1.01)	0.063
Diabetes	Yes	1.31 (1.03, 1.67)	0.032	1.56 (1.12, 2.17)	0.009	1.00 (0.73, 1.38)	0.980	1.57 (1.16, 2.14)	0.004	1.31 (0.94, 1.81)	0.108
Dyslipidemia	Yes	0.85 (0.68, 1.05)	0.130	0.84 (0.6, 1.17)	0.308	0.83 (0.61, 1.12)	0.220	1.01 (0.78, 1.31)	0.922	0.80 (0.60, 1.08)	0.147
Hypertension	Yes	1.06 (0.87, 1.28)	0.574	1.10 (0.82, 1.46)	0.525	0.98 (0.77, 1.25)	0.875	1.26 (0.97, 1.63)	0.086	1.36 (1.05, 1.75)	0.019
eGFR, ml/min/1.73m^2	eGFR≥ 60	1.31 (0.73, 2.38)	0.365	1.30 (0.60, 2.78)	0.513	1.35 (0.54, 3.45)	0.523	1.92 (0.78, 4.76)	0.160	1.61 (0.85, 3.03)	0.146
Hyperuricemia[‡]	Yes	1.14 (0.84, 1.53)	0.400	1.08 (0.75, 1.56)	0.664	1.34 (0.83, 2.16)	0.226	0.77 (0.55, 1.08)	0.133	1.85 (1.12, 3.09)	0.018

*Defined as consuming alcohol more than two or three times per week in the past year before the interview.

†Defined as history of longer than 3 years of exposure to loud noise at work. Loud noise means that the noise in the workplace is so loud that conversation is impossible.

‡Defined as > 7 mg/dL in males and > 6 mg/dL in females.

OR, odds ratio; BMI, body mass index; eGFR, estimated glomerular filtration rate.

Supplementary Table S2. Multivariable logistic regression analysis to predict risk of low/mid frequency hearing impairment in the Korean adult population according to age and sex.

Variable	category	Total		Male		Female		Age < 60 years		Age ≥ 60 years	
		Weighted OR (95% CI)	P-value	Weighted OR (95% CI)	P -alue	Weighted OR (95% CI)	P-value	Weighted OR (95% CI)	P-value	Weighted OR (95% CI)	P-value
Age, years		1.14 (1.12, 1.16)	< 0.001	1.13 (1.11, 1.15)	< 0.001	1.14 (1.11, 1.17)	< 0.001				
Sex (reference : male)	Female	0.70 (0.49, 0.98)	0.040					0.51 (0.28, 0.92)	0.026	0.59 (0.43, 0.82)	0.002
Education level (reference : Elementary)			< 0.001		0.018		0.003		< 0.001		< 0.001
	Middle school	0.67 (0.48, 0.93)	0.018	0.96 (0.57, 1.62)	0.876	0.53 (0.35, 0.81)	0.003	0.62 (0.25, 1.54)	0.302	0.50 (0.35, 0.70)	< 0.001
	High school	0.75 (0.55, 1.03)	0.082	1.03 (0.65, 1.63)	0.912	0.61 (0.40, 0.94)	0.024	0.63 (0.24, 1.62)	0.338	0.39 (0.29, 0.52)	< 0.001
	University	0.42 (0.28, 0.62)	< 0.001	0.56 (0.34, 0.92)	0.023	0.31 (0.16, 0.61)	0.001	0.18 (0.07, 0.50)	0.001	0.29 (0.20, 0.41)	< 0.001
Current smoking (reference : never smoking)			0.480		0.672		0.272		0.648		0.473
	Ex-smoker	0.89 (0.61, 1.29)	0.544	0.88 (0.57, 1.35)	0.554	0.58 (0.22, 1.52)	0.266	0.93 (0.48, 1.80)	0.837	0.85 (0.58, 1.24)	0.401
	Current smoker	1.13 (0.70, 1.81)	0.626	1.02 (0.61, 1.73)	0.930	1.60 (0.69, 3.71)	0.278	0.73 (0.35, 1.51)	0.396	0.75 (0.47, 1.19)	0.230
Heavy alcohol use*	Yes	1.19 (0.86, 1.66)	0.298	1.28 (0.89, 1.84)	0.184	0.84 (0.42, 1.66)	0.615	1.58 (0.93, 2.70)	0.093	0.78 (0.56, 1.09)	0.145
Occupational noise exposure†	Yes	1.93 (1.49, 2.50)	< 0.001	2.37 (1.68, 3.35)	< 0.001	1.31 (0.86, 2)	0.212	2.33 (1.34, 4.05)	0.003	1.29 (1.00, 1.67)	0.051
Body mass index (kg/m^2)	BMI≥25	0.95 (0.76, 1.20)	0.683	0.99 (0.68, 1.42)	0.942	0.88 (0.66, 1.18)	0.408	0.93 (0.51, 1.68)	0.805	0.79 (0.63, 0.98)	0.031
Diabetes	Yes	1.02 (0.79, 1.30)	0.889	0.95 (0.67, 1.34)	0.776	1.02 (0.71, 1.45)	0.925	1.22 (0.65, 2.27)	0.541	1.08 (0.85, 1.38)	0.513
Dyslipidemia	Yes	1.06 (0.83, 1.34)	0.642	1.22 (0.85, 1.74)	0.275	0.93 (0.71, 1.22)	0.606	1.02 (0.59, 1.75)	0.945	1.02 (0.79, 1.30)	0.901
Hypertension	Yes	1.00 (0.77, 1.29)	0.983	0.94 (0.67, 1.33)	0.739	1.00 (0.72, 1.38)	0.987	1.23 (0.67, 2.27)	0.499	1.26 (0.99, 1.60)	0.058
eGFR, ml/min/1.73m^2	eGFR≥ 60	1.10 (0.73, 1.67)	0.657	1.03 (0.57, 1.89)	0.913	1.15 (0.63, 2.13)	0.646	4.55 (0.96, 20.00)	0.057	1.43 (1.00, 2.08)	0.051
Hyperuricemia	Yes	0.99 (0.73, 1.35)	0.959	0.88 (0.58, 1.34)	0.559	1.16 (0.70, 1.92)	0.573	0.79 (0.37, 1.68)	0.537	1.04 (0.75, 1.45)	0.795

*Defined as consuming alcohol more than two or three times per week in the past year before the interview.

†Defined as history of longer than 3 years of exposure to loud noise at work. Loud noise means that the noise in the workplace is so loud that conversation is impossible.

‡Defined as > 7 mg/dL in males and > 6 mg/dL in females.

OR, odds ratio; BMI, body mass index; eGFR, estimated glomerular filtration rate.
